# Post‐operative minimal residual disease models to study metastatic relapse in soft‐tissue sarcoma patient‐derived xenografts

**DOI:** 10.1002/ctm2.1290

**Published:** 2023-06-06

**Authors:** Suzanne Fischer, David Creytens, Sofie De Geyter, Elly De Vlieghere, Piet Pattyn, Sarah‐Lee Bekaert, Kaat Durinck, Nadine Van Roy, An Hendrix, Lore Lapeire, Gwen Sys, Olivier De Wever

**Affiliations:** ^1^ Laboratory of Experimental Cancer Research Department of Human Structure and Repair Ghent University Ghent Belgium; ^2^ Department of Gastro‐Intestinal Surgery Ghent University Hospital Ghent Belgium; ^3^ Cancer Research Institute Ghent Ghent Belgium; ^4^ Department of Pathology Ghent University Hospital Ghent Belgium; ^5^ Department of Biomolecular Medicine Ghent University Ghent Belgium; ^6^ Pediatric Precision Oncology Lab Ghent University Ghent Belgium; ^7^ Centre for Medical Genetics Ghent University Hospital Ghent Belgium; ^8^ Department of Medical Oncology Ghent University Hospital Ghent Belgium; ^9^ Department of Orthopedics and Traumatology Ghent University Hospital Ghent Belgium

Dear Editor,

Soft‐tissue sarcomas (STS) are rare, heterogeneous cancers comprising 1% of adult and 15% of paediatric malignancies. Despite optimal treatment, 50%−80% of patients metastasize, even when they attain a status of minimal residual disease (MRD). MRD is achieved through multimodal treatment involving primary tumour resection with wide negative margins. In metastatic setting, systemic therapies are palliative and response rates are low (15%–20%).^1^, ^2^ As a result of these poor outcome data, there is a strong need for translationally relevant patient‐derived models.

Patient‐derived xenografts (PDX) are used to investigate novel therapies and guide personalized treatment response.^3^ Yet, many PDX models do not reflect the clinical behaviour of human tumours.^4^ For STS, commonly used PDX still fail to predict the clinical efficacy of (novel) drugs and indeed, MRD status and subsequent metastatic progression have been poorly modelled in STS‐PDX^5^ (Table S1). Moreover, currently available STS‐PDX models have not been comparatively assessed. In this study we aimed to address this translational gap by creating PDX that mimic MRD status (MRD‐PDX), followed by metastatic relapse and examine the most appropriate model. We first engrafted tumour tissue derived from five high‐grade STS patients (Table S2) and resected the subsequent primary tumour at a size of 250‐450 mm^3^ using limb amputation to obtain negative surgical margins,^6^ similar to the patient's treatment (Figure 1A). The impact of the site of transplantation (orthotopic [O‐PDX] vs subcutaneous [SC‐PDX]) and immunodeficiency status of the host animal (NOD scid gamma [NSG] vs Swiss nu/nu mice) on primary tumour growth, MRD and disease progression (local recurrence and metastasis) were directly compared for four patients (Figure 1B). PDX were followed up to 1 year after tumour resection. MRI monitored primary tumour growth, MRD and metastatic relapse (Figure 1A). Histopathology and copy number variation (CNV) sequencing evaluated tumour characteristics.

**FIGURE 1:**
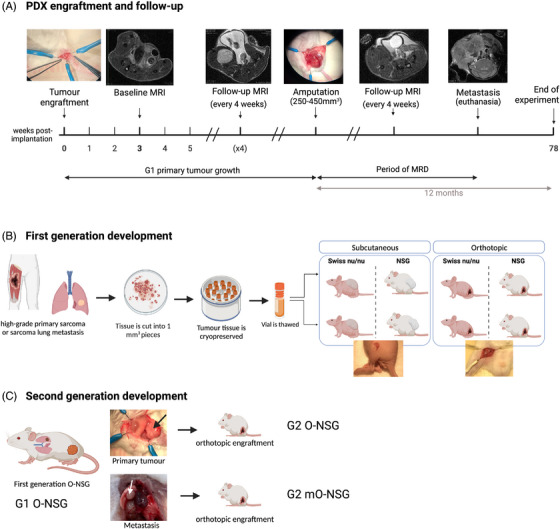
Development protocol of the MRD‐PDX model. (A) Animals were followed until metastasis, or until 12 months post primary tumour resection if no metastases developed, or until 18 months (78 weeks) post tumour implantation if no primary tumour developed. MRD, minimal residual disease. (B) Schematic visualization of the comparative assessment of mouse strains and implantation methods for the development of sarcoma patient‐derived xenografts. NSG, NOD scid gamma; orthotopic, orthotopic tumour tissue piece engraftment; Subcutaneous, subcutaneous tumour tissue piece engraftment; . (C) Schematic visualization of the development of second‐generation orthotopic NSG, based on either first‐generation primary tumours or first‐generation metastasis. O‐NSG, orthotopic engraftment of sarcoma tissue in NSG mice. Black arrow shows primary tumour growth, white arrow lung metastasis.

Orthotopic tumours had a range of macroscopic and radiologic differences compared to subcutaneous STS, including irregular shape, heterogeneity, lack of circumscription, increased vascularization and unclear margins with the surrounding tissue (Figure 2A). Histologically, orthotopic tumours were uncapsulated, showed invasion of the surrounding tissue, vascular and neural encasement and necrosis. Alpha smooth muscle actin (αSMA), which can be indicative for cancer‐associated fibroblasts,^7^ was positive homogenously throughout orthotopic tumours, and only peripherally in subcutaneous tumours derived from the same patient (Figure 2B).

**FIGURE 2:**
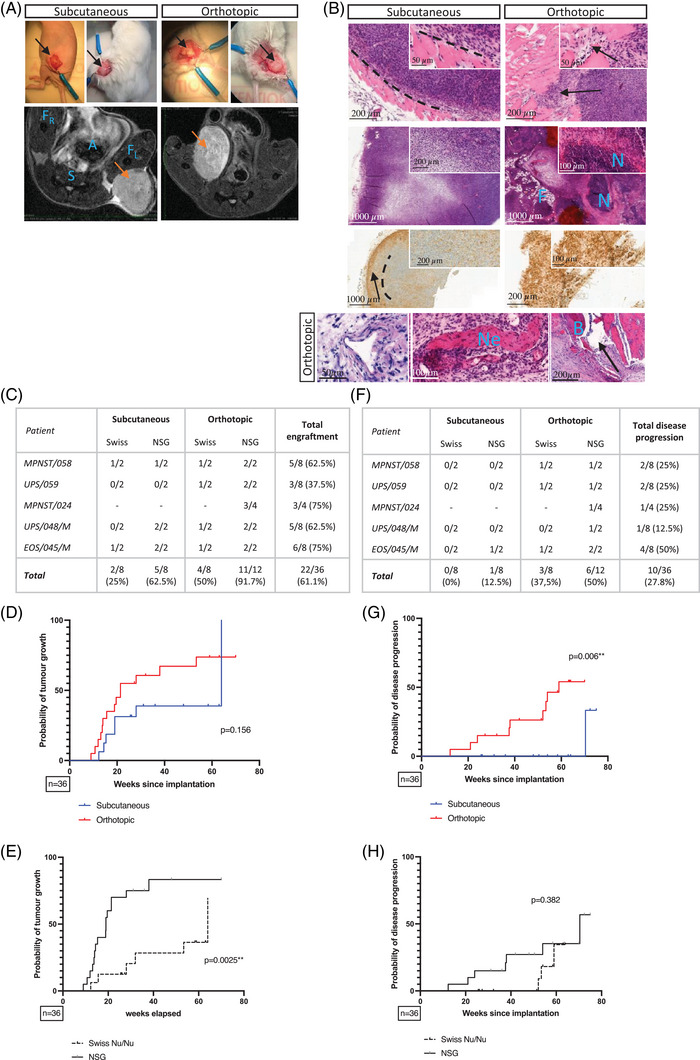
Impact of engraftment site and mouse strain on primary tumour growth and disease progression. (A) Macroscopical and radiological comparison of subcutaneous and orthotopic tumours. Black arrows indicate primary sarcoma in the upper panels. The lower panels show axial T2‐weighted MRI images, the orange arrows indicate primary sarcoma growth. A, abdomen; F_L_, left femur; F_R_, right femur; S, spine. (B) Histological comparison of subcutaneous and orthotopic tumours by haematoxylin and eosin and αSMA staining. First row, left panel: the dotted line indicates the border of the tumour, which does not infiltrate the surrounding tissue. First row, right panel: black arrow indicates a finger‐like infiltrating pattern of the tumour in the surrounding muscular tissue. The second row shows the difference in heterogeneity of the tissue with large areas of necrosis and enveloped fat tissue in the orthotopic implantation model. F, fat tissue; N, necrosis. The third row shows the difference in αSMA staining pattern. The arrow in the left panel indicates a border of positivity as further indicated by the dotted line. In the right panel, the whole specimen is positive. The fourth row shows tumour microenvironment characteristics uniquely observed in orthotopic samples. Left panel: blood vessel encasement. Middle panel: nerve encasement (Ne, nerve). Right panel: infiltrating tumour tissue (arrow) in the bone. (C) Tumour take ratios (the proportion of animals showing tumour growth) for PDX from patients MPNST/058 (primary malignant peripheral nerve sheath tumour), UPS/059 (primary undifferentiated pleomorphic sarcoma), MPNST/024 (primary MPNST), UPS/048/M (lung metastasis of an UPS) and EOS/045/M (lung metastasis of an extra‐skeletal osteosarcoma). Kaplan–Meier graphs indicating the probability of primary tumour growth comparing (D) implantation site or (E) mouse strain, where ‘event’ is detection of tumour growth and censored when no tumour was detected at time of necropsy. (F) Disease progression ratios for all included patients (the proportion of animals showing metastasis and/or local recurrence). Kaplan–Meier curves indicating probability of disease progression comparing (G) implantation site or (H) mouse strain where ‘event’ is detection of metastasis and censored when no metastasis were detected at time of necropsy.

Overall engraftment ratio for all models was 61.1% (25%–91.7%) and was highest in O‐NSG (Figure 2C). Probability of tumour growth (Kaplan–Meier method) showed no significant difference in engraftment efficiency between SC‐ and O‐PDX (*p* = .156, Figure 2D), but was significantly more efficient in NSG, compared to Swiss nu/nu (*p* = .0025, Figure 2E). Overall disease progression ratio for all models was 27.8% (0%–50%) and was again highest in O‐NSG (Figure 2F). Probability of disease progression was significantly higher in O‐PDX, compared to SC‐PDX (*p* = .006, Figure 2G), but was not significantly impacted by mouse strain (*p* = .382, Figure 2H). PDX tumour volume at time of resection was investigated as confounding factor for disease progression but was not significantly different between PDX that did and did not metastasize (*p* = .351). Resection margins were negative in all animals.

The O‐NSG model was selected as the most applicable and usable as MRD‐PDX due to the high probability of tumour take and disease progression, with an overall MRD period of 14.61 ± 6.09 weeks (Figure 3). In contrast, only 1 out of 16 SC‐PDX showed disease progression (local recurrence). This model was derived from a patient lung metastasis (EOS/045/M) and had an MRD period of 51.4 weeks. Although disease progression in O‐Swiss did not significantly differ from O‐NSG, we do not support this as MRD‐PDX model. First, the take ratio is lower, which reduces the usability. Second, out of the three O‐Swiss that showed metastases, only one had been preceded by a primary tumour (MRD period of 36.5 weeks). The two others developed metastases without clinically and radiologically detectable primary tumour growth, which was never the case in O‐NSG. Our observations add to the evidence supporting that the microenvironment at the implantation site determines tumour behaviour.^4^

**FIGURE 3:**
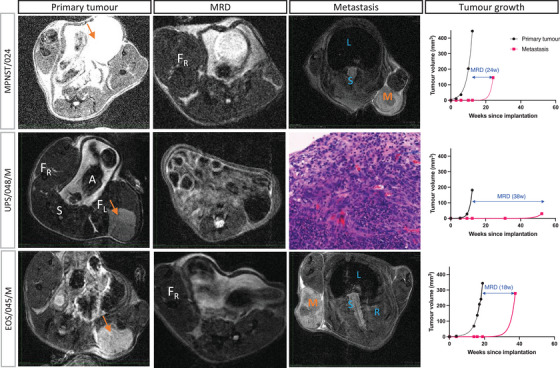
Minimal residual disease (MRD) in orthotopic sarcoma patient‐derived xenografts, exemplified for three cases. T2‐weighted axial MRI images of primary orthotopic tumours (first column, orange arrows), of MRD status (post‐amputation status, second column) and metastasis (third column, M, metastasis) for patients MPNST/024, UPS/048/M and EOS/045/M. A, abdomen; F_L_, left femur; F_R_, right femur; L, lungs; M, metastasis; R, ribs; S, spine, . Metastases for UPS/048/M were located in the head of the pancreas and were not clearly visible on MRI. A haematoxylin–eosin image of the metastasis is shown on a 20× magnification (third column, second row). Fourth column: nonlinear fitted growth curves of the primary tumour and post‐amputation metastasis. Double arrow indicates MRD status with number of weeks (*w*) between brackets.

Based on these results, we developed second‐generation O‐NSG by implanting either primary tumour (G2 O‐NSG) or metastasis tissue (G2 mO‐NSG) derived from first‐generation (G1) O‐NSG (Figure 1C). Take ratio was 100% for both G2 and tumour growth was significantly faster compared to G1 (Figure 4A), as observed in other tumours.^8^ G2 mO‐NSG exhibited a more aggressive phenotype with faster‐developing metastases (MRD period of 2.6 ± .084 weeks) (Figure 4B). Similar metastatic patterns were observed in G1, G2 mO‐NSG and patients (Table S3, Supporting Information).

**FIGURE 4:**
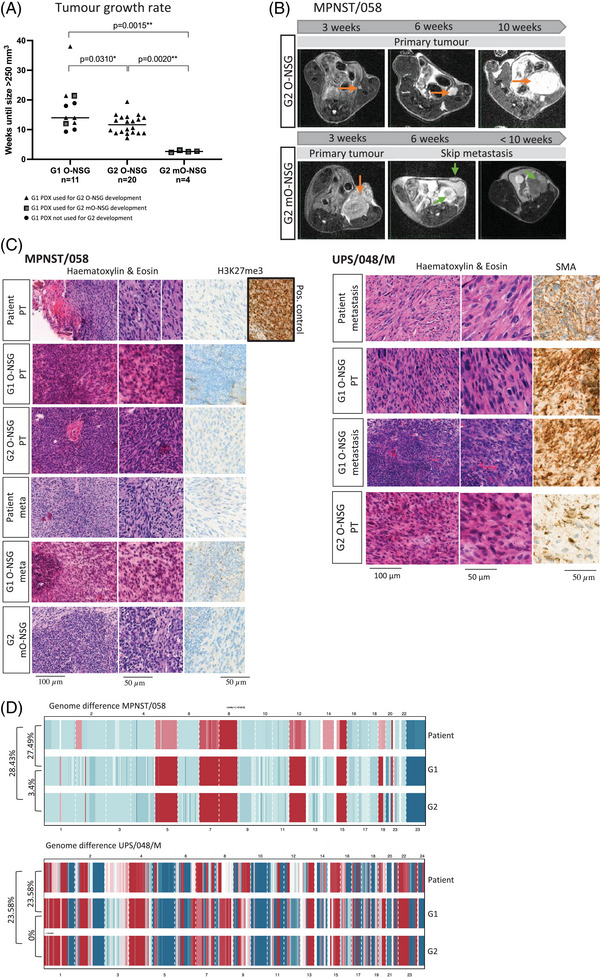
Second‐generation development. (A) Difference in tumour growth rate between first‐generation and second‐generation orthotopic PDX. G1 O‐NSG, first‐generation PDX developed by orthotopic engraftment of patient‐derived tumour tissue in NSG mice; G2 O‐NSG, second‐generation PDX developed by orthotopic engraftment of G1 O‐NSG primary tumour tissue in NSG mice; G2 mO‐NSG, second‐generation PDX developed by orthotopic engraftment of G1 O‐NSG metastasis in NSG mice. The symbol legend indicates which first‐generation PDX were used to further develop second‐generation PDX (G2 O‐NSG and G2 mO‐NSG). Asterisk indicate statistical significance, *p < .05, **p < .01. (B) Overview of orthotopic tumour growth in second‐generation PDX for MPNST/058 on axial T2‐weighted MRI images. Orange arrows indicate primary tumour growth. Green arrows indicate metastatic lesions. (C) Illustration of the comparison of histology between patient and first‐ and second‐generation PDX tumours on haematoxylin and eosin staining, as well as immunohistochemical staining: H3K27me3 for malignant peripheral nerve sheath tumours and αSMA staining for undifferentiated pleomorphic sarcoma. Loss of H3K27me3 is diagnostic for malignant peripheral nerve sheath tumours, all PDX generations of the MPNST/058 example remain negative. A positive control is included as comparison. meta, metastasis; PT, primary tumour; . (D) Illustration of the comparison of copy number variations (CNV) between patient primary tumour (PT), and primary tumours in first‐ (G1) and second (G2)‐generation orthotopic NSG mice for MPNST/058 and UPS/048/M. Genome differences (%) are indicated at the left side of the illustration.

The histological phenotype of STS (atypical, pleomorphic spindle cells with abundant mitotic figures) and immunohistochemistry pattern were maintained throughout the different PDX passages, including PDX metastases (Figure 4C). Remarkably, histologic architecture was highly similar between patient and PDX metastases. The genome difference, characterized by CNV, was only 0%–3.4% between G1 and G2 O‐NSG primary tumours, suggesting that epigenetic or transcriptional adaptations drive the higher efficiency rates of the next generations. As expected from previous reports,^9^ discrete changes in CNV were observed between the patient's primary tumour sample and PDX passages (genome difference 23.58%–28.43%) (Figure 4D).

Potential applications of MRD‐PDX include investigating the impact of surgery on disease progression, biomarker studies to determine the detection limit of MRD; evaluating therapies maintaining MRD‐status and modelling metastasis‐targeted therapies.^4^ Unfortunately, due to the absence of an intact immune system, evaluation of anti‐PD1 therapy, which looks promising for some STS subtypes,^10^ is not possible. Although primary tumour‐derived MRD‐PDX developed metastasis faster than the corresponding patients in this study, MRD‐PDX require a long observation time, and as it remains impossible to predict which high‐grade patients are at risk for metastasis, this hampers the usability in personalized medicine. Due to the heterogeneity of STS, a one‐size‐for‐all solution cannot be expected, and we strongly believe that these models are a valuable, but complementary addition to other available preclinical models. Further validation in larger STS MRD‐PDX cohorts will be an important contribution to the current available data.

In conclusion, MRD‐PDX developed by orthotopic implantation in NSG mice followed by surgical removal of the primary tumour accurately simulate the clinical behaviour of STS. This model holds MRD‐specific benefits and should not be neglected when evaluating novel therapies.

## CONFLICT OF INTEREST STATEMENT

The authors declare no conflicts of interest.

## Supporting information

Supporting information

Supporting information

Supporting information

Supporting information

Supporting information
