# Supplementary material for: Post‐operative minimal residual disease models to study metastatic relapse in soft‐tissue sarcoma patient‐derived xenografts
Source: Clin Transl Med. 2023 Jun 6;13(6):e1290. doi: 10.1002/ctm2.1290 (PMC10244893; doi:10.1002/ctm2.1290)
Supplement: Supplementary file 1 — Supporting information [file CTM2-13-e1290-s002.docx]

## Supplementary methods

### Patient selection and ethics approval

The collection and processing of human high-grade soft tissue sarcoma tissue was approved by the ethical committee of Ghent University Hospital (EC 2018/0080). Patient Informed Consent Forms were obtained for the approval of tissue harvesting, storage and establishment of patient-derived xenografts.

Five patients were selected, three patients presented with a primary tumour: two high-grade malignant peripheral nerve sheath tumours (MPNST/058, MPNST/024) and a high-grade undifferentiated pleomorphic sarcoma (UPS/059), and two patients presented with lung metastasis: a high-grade undifferentiated pleomorphic sarcoma (UPS/048/M) and a high-grade extraskeletal osteosarcoma (EOS/045/M).

### Tissue sampling

Tumour samples for PDX development were obtained during sarcoma biopsy or resection (Table S2) and were immediately processed in the operating theatre under sterile conditions. The processing was kept strictly separate from the patient’s surgical field. Samples were minced with a sterile scalpel into tumour pieces of 1-2 mm3 and stored in cryovials containing 1 ml freezing medium (90% heat-inactivated fetal bovine serum (FBS, cat. no. ATCC-30-2030, Waltham, MA, ThermoFischer), 10% DMSO (Novolab, Belgium)) at -80°C, using a Cell Freezing Container (Corning, Inc., Corning, NY, USA). Transfer time between the operating theatre and storage at -80°C was less than 15 minutes. A minimum of 3 vials each containing 4-6 tumour pieces were obtained for every patient. For long term storage, samples were cryopreserved in liquid nitrogen.

### Experimental design

*Comparative study for set-up of first generation (G1) PDX models:* four patients were selected for this pilot study: MPNST/058, UPS/059, UPS/048/M and EOS/045/M. For every patient, tumour tissue was implanted in a total of 8 mice at the left or right lower limb of which 4 Swiss nu/nu mice: 2 subcutaneously and 2 orthotopically (G1 SC-Swiss and G1 O-Swiss), and 4 NOD-SCID-IL2γ−/− (NSG) mice: 2 subcutaneously and 2 orthotopically (G1 SC-NSG and G1 O-NSG) (Figure 1B). After implantation, animals were followed clinically and radiologically. Both SC- and O-PDX were imaged with a 7 Tesla magnetic resonance imaging (MRI) Scanner for Small Animal Imaging Research (BioSpin Pharmascan 70/16, Bruker, Belgium, at Infinity, Ghent University, Belgium) in T2 sequence to assess tumour growth. MRI was performed under general anaesthesia (isoflurane, induction 4%, maintenance 2-3%, flow 0,8-1L/min). Tumour volumes were calculated based on MRI volume rendering using Horos^TM^ (Annapolis, MD USA). Animals were initially scanned at 3 weeks post-implantation and were then scanned monthly, or earlier if faster tumour growth occurred. To achieve negative resection margins and thus simulate the human clinical setting faithfully, the hindlimb was amputated when tumours reached a size 250-450mm^3^, allowing angiogenesis and dissemination^1^, while at the same time preventing morbidity associated with the primary tumour^2^. A section of the resection plane was made and embedded in paraffin to assess resection margins. Animals were re-imaged 3 weeks after amputation and then monthly to monitor local recurrence. Animals were weighed and clinically assessed twice weekly for physical signs of metastasis. When either local recurrence or metastasis occurred, when animals lost weight rapidly, or when a minimum follow-up of 18 months post-implantation or 1-year post-amputation was reached, animals were euthanatized by cervical dislocation (Figure 1A). Lungs, liver, spleen, salivary glands, lymph nodes, visible metastases and local recurrence if present, surrounding tissue of primary tumour implantation location and a new intersection of the resection margin to detect remaining tumour cells, were harvested at necropsy.

Tissue samples of the primary, local recurrence and metastasis were again cut into 1-2 mm^3^ pieces and stored as described above. Remaining fragments were formaldehyde fixed and used for histopathology.

*Development of second generation (G2) PDX:* primary tumour tissue was harvested from G1 O-NSG during amputation. Metastases were harvested at necropsy of G1 O-NSG, if present. Both primary tumour and metastases were used to set up G2 PDX by orthotopic implantation in NSG mice. For all patients, *G2 O-NSG* models were developed by implanting G1 O-NSG primary tumour tissue (1-2 mm^3^) in 4 G2 NSG mice. For 2 patients (MPNST/058, UPS/059), *G2 mO-NSG* models were developed by orthotopically implanting G1 O-NSG spontaneous metastasis tissue (1-2 mm^3^) in 2 NSG mice. Animals were assessed both clinically and radiologically, analogous to G1, using the same timeline.

*Expansion of the MRD-PDX biobank:* a new patient (MPNST/024) was included for the development of MRD-PDX. Tissue was implanted in 4 G1 O-NSG and subsequently in 4 G2 O-NSG mice.

### Subcutaneous and orthotopic tissue engraftment

The first generation of PDX was initiated within 1 year after sample collection. Cryopreserved tumour tissue was thawed by diluting the freezing medium 1:50 with DMEM 10% FBS supplemented with penicillin and streptomycin at 37°C^3^. A tumour tissue piece of 1-2 mm^3^ was either implanted subcutaneously or orthotopically on the left or right lower limb. Incision to closure time was 5 minutes maximum. All surgeries were performed under sterile conditions. Animals were randomized using Microsoft Excel. Animals were anesthetized with isoflurane (induction 4%, maintenance 2-3%, flow 0,8-1L/min). The surgical region was disinfected with chlorhexidine 0,2%. For NSG models, hair was clipped short at the surgical region before disinfection.

*Subcutaneous implantation:* a small incision in the skin (max. 5 mm) was made with surgical scissors on the lateral region of the hindlimb and a small pocket was created medially from the incision right under the skin by blunt dissection. A tumour fragment was placed in the pocket and the incision was closed with Vicryl 6.0 running suture (Ethicon, Raritan, NJ, USA) and 1 drop of Vetbond (3M, Saint Paul, MN, USA).

*Orthotopic implantation of UPS and EOS:*  animals were positioned on their side, a small incision in the skin (5 mm) was made with surgical scissors on the lateral region of the hindlimb. The underlying muscle (quadriceps femoris) was split, and a pocket was created within the muscle. A tumour fragment was slid into the pocket, and the muscle pocket was closed with 1 stich of coated Vicryl 8.0 (Ethicon). The incision was closed with Vicryl 6.0 running suture and 1 drop of Vetbond to prevent the animals from scratching the stiches.

*Orthotopic implantation of MPNST:* the animals were positioned supine, a small incision 3 mm caudal from the inguinal ligament was made in the skin (5 mm) with surgical scissors. The underlying fascia was dissected until the femoral nerve could be clearly visualised. The nerve was loosened from the surrounding tissue and a pocket was created deep from the femoral nerve to position the tissue fragment. The surrounding muscle was used to enclose the pocket around the nerve with 1 stich of Vicryl 8.0. The skin was closed as described previously.

### Hindlimb amputation to achieve minimal residual disease

Animals were anesthetized and prepared as described above. Before the start of the surgery, animals received ketoprofen 2-5mg/kg intraperitoneally for analgesia and 200μl saline solution to compensate for potential blood loss. Animals were supine positioned. An inguinal incision (8 mm) was made with surgical scissors. Blunt wound retractors were used to open the surgical area. The skin was loosened from the muscle with both blunt and sharp dissection. The neurovascular bundle (femoral nerve, artery, and vein) was located exiting the peritoneum and ligated grossly with 2 proximal and 1 distal suture (Vicryl 8.0) as far as possible from the tumour. The surrounding muscle tissue was bluntly dissected from the skin and then transected with scissors. The femur was cut with large, sharp surgical scissors uniquely used for bone transection. A skin flap was created. The remaining muscle tissue was closed over the stump to prevent bleeding from the bone (Vicryl 8.0) and the skin was closed with a simple running suture (Vicryl 6.0) and multiple drops of Vetbond. Recovery from amputation was fast and within 30 minutes the animals were behaving as usual, without need for segregation. Food and water intake was as before.

## Supplementary references

1. Krishnan, K., Khanna, C., Helman Lee J, and Maryland, B. (2005). The biology of metastases in pediatric sarcomas. The Cancer Journal *11*, 306–313.

2. Khanna, C., Prehn, J., Yeung, C., Caylor, J., Tsokos, M., and Helman, & L. (2000). An orthotopic model of murine osteosarcoma with clonally related variants differing in pulmonary metastatic potential. Clin Exp Metastasis *18*, 261–271.

3. Linnebacher, M., Maletzki, C., Ostwald, C., Klier, U., Krohn, M., Klar, E., and Prall, F. (2010). Cryopreservation of human colorectal carcinomas prior to xenografting. BMC Cancer *10*, 1471–2407.
