# Supplementary material for: Post‐operative minimal residual disease models to study metastatic relapse in soft‐tissue sarcoma patient‐derived xenografts
Source: Clin Transl Med. 2023 Jun 6;13(6):e1290. doi: 10.1002/ctm2.1290 (PMC10244893; doi:10.1002/ctm2.1290)
Supplement: Supplementary file 2 — Supporting information [file CTM2-13-e1290-s005.docx]

**Supplemental table 1. Patient-derived xenografts in sarcoma research.**

*Location* is anatomical location of the engrafted specimen. *Method* is implantation or injection. *Specimen* is the engrafted substrate. *Follow-up* is the time the animals were clinically observed, until euthanasia. *Metastasis* attributes the observation of metastasis after tumour implantation. *Not reported*: the authors of the article don’t mention the presence nor absence of metastasis, *not observed*: the authors of the article did not observe any metastasis. This table is specifically focused on xenografts using patient-derived tumor fragments or cell suspensions without in vitro subculture. We did not include xenograft models using established cell lines (which show culture specific selections and adaptations).

| Location | Method | Sarcoma subtype | Specimen | Mouse strain | Details | Follow-up | Metastasis | Ref. |
| --- | --- | --- | --- | --- | --- | --- | --- | --- |
| Subcutaneous | Implantation | Osteosarcoma | Primary tumour tissue | NSG  RGKO |  | 4 weeks | Not reported  Not reported/not detected | ^4,5^ |
|  |  |  | Patient-derived metastatis | Atymic Nu/Nu  NSG  RGKO |  | 4-52 weeks |  | ^4–7^ |
|  |  |  | Patient-derived pleural effusion | NSG |  | 20 weeks | No metastasis | ^7^ |
|  |  | Ewing Sarcoma | Primary tumour tissue | Atymic nu/nu NSG  RGKO |  | 4-52 weeks | Not reported | ^4,5,8^ |
|  |  |  | Patient-derived metastasis | NSG  RGKO |  | 4 weeks |  | ^4,5^ |
|  |  |  | Unspecified | NSG |  | 20 weeks | Not detected | ^7^ |
|  |  | Extraskeletal osteosarcoma | Primary tumour tissue | Nu/Nu NMRI  NSG  RGKO |  | 4 weeks | Not reported | ^4,9^ |
|  |  |  | Patient-derived metastasis | NSG  RGKO |  | 4 weeks |  | ^4^ |
|  |  | Undifferentiated pleomorphic sarcoma | Tumor tissue, not specified | Ragγ2C-/- |  | 5 weeks | Not reported | ^10,11^ |
|  |  |  | Primary tumour tissue | Athymic nu/nu | Implantation of 4 fragments | 8 weeks | Not reported | ^8,12^ |
|  |  |  |  | Nu/nu NMRI |  | Unspecified |  | ^9^ |
|  |  | Undifferentiated small round cell sarcoma | Tumour tissue, not specified | NOG |  | 6 weeks | Not reported | ^13^ |
|  |  |  | Primary tumour tissue | Nu/Nu NMRI |  | Unspecified | Not reported | ^9^ |
|  |  | Dedifferentiated liposarcoma | Primary tumour tissue | Nu/nu NMRI | Bilateral subcutaneous implantation | 3 weeks | Not reported | ^8,9,12,14^ |
|  |  |  |  | Athymic nu/nu | Implantation of 4 tumour fragments | 4 months |  |  |
|  |  |  | Patient-derived metastasis | Nu/nu NMRI | Bilateral subcutaneous implantation | 3 weeks | Not reported | ^14^ |
|  |  | Synovial sarcoma | Primary tumour tissue | Athymic nu/nu  Nu/nu NMRI | Bilateral subcutaneous implantation | 3 weeks – 50 days | Not reported | ^8,9,14^ |
|  |  |  | Patient-derived metastasis | Nu/nu NMRI | Bilateral subcutaneous | 3 weeks | Not reported | ^14^ |
|  |  | Myxofibrosarcoma | Primary tumour tissue | Athymic nu/nu | Implantation of 4 fragments | 3 months | Not reported | ^12^ |
|  |  |  |  | Nu/nu NMRI |  | Not specified |  | ^9^ |
|  |  | Leiomyosarcoma | Primary tumour tissue | Athymic nu/nu  Nu/Nu MRI |  | 50 days | Not reported | ^8,9^ |
|  |  | Chondrosarcoma | Primary tumour tissue | Athymic nu/nu |  | 50 days | Not reported | ^8,9^ |
|  |  | Fibrosarcoma | Primary tumour tissue | Athymic nu/nu |  | 50 days | Not reported | ^8,9^ |
|  |  | Rhabdomyosarcoma | Primary tumour tissue | Athymic nu/nu |  | 50 days | Not reported | ^8,9^ |
|  |  | Malignant Peripheral Nerve Sheath Tumour | Primary tumour tissue | Athymic nu/nu |  | Unspecified | Not reported | ^9^ |
|  |  | Pulmonary artery intima sarcoma | Primary tumour tissue | Athymic nu/nu |  | Unspecified | Not reported | ^9^ |
|  |  | Soft tissue sarcoma of the retroperitoneum | Primary tumour tissue | Athymic nu/nu | Tumour resection and follow-up for local recurrence | 8 weeks | No local recurrence | ^15^ |
| Orthotopic | Injection | Osteosarcoma | Primary tumour tissue | NSG  Foxn1/nu | G1 subcutaneous implantation, G2 Intra-tibial injection single cell suspension | 2-3 months | Lung metastasis | ^16^ |
|  |  |  |  | G1 NSG G2 Athymic nu/nu | G1 intra-femoral injection of a patient-derived single cell suspension | 140 days | Not reported | ^17^ |
|  |  | Ewing sarcoma | Patient-derived single cell suspension | G1 NSG G2 Athymic nu/nu | Intra-femoral injection of a patient-derived single cell suspension | 140 days | Not reported | ^17^ |
|  |  | Rhabdomyosarcoma | Patient-derived single cell suspension | G1 NSG G2 Athymic nu/nu | Intramuscular injection of a patient-derived single cell suspension | 140 days | Not reported | ^17^ |
|  |  | Undifferentiated pleomorphic sarcoma | Patient-derived single cell suspension | G1 NSG G2 Athymic nu/nu | Intramuscular injection of a patient-derived single cell suspension | 140 days | Not reported | ^17^ |
|  | Implantation | Osteosarcoma | Primary tumour | Athymic nu/nu  NSG | G1 subcutaneous implantation, G2 orthotopic implantation | 15 days after size > 100mm^3^ | Not reported | ^18^ |
|  |  |  | Lung metastasis | Athymic nu/nu | G1 subcutaneous implantation, G2 orthotopic implantation in the femur | 15 days after size > 100mm^3^ | Not reported | ^19^ |
|  |  |  |  |  | G1 subcutaneous implantation, G2 orthotopic implantation in the lung  Pre-tibial space, limb amputation when tumour reached 1,5cm diameter | 14 days  20 weeks | Not reported  Pulmonary and intra-abdominal metastases after amputation | ^20,21^ |
|  |  |  |  |  |  |  |  | ^7,22^ |
|  |  |  | Pleural effusion | NSG | Pre-tibial space, limb amputation when tumour reached 1,5cm diameter | 20 weeks | Axillary nodes, kidney, ovary, peritoneum after amputation | ^7,22^ |
|  |  | Ewing sarcoma | Primary tumour tissue | Athymic nu/nu | G1 subcutaneous implantation, G2 orthotopic implantation | 15 days after size > 60mm^3^ | Not reported | ^7,22–29^ |
|  |  |  | Unspecified | NSG | Pre-tibial space, limb amputation when tumour reached 1,5cm diameter | 20 weeks | Lung metastasis after amputation | ^7,22^ |
|  |  | Soft tissue sarcoma of the retroperitoneum | Primary tumour tissue | Athymic nu/nu | G1 orthotopic implantation at the kidney | 8 weeks | Not reported | ^15^ |
|  |  | Undifferentiated pleomorphic sarcoma | Primary tumour tissue | Athymic nu/nu | G1 subcutaneous implantation, G2 orthotopic implantation | 15 days after size > 100mm^3^ | Not reported | ^30–43^ |
|  |  |  |  | RFP expressing transgenic nude mice | G1 subcutaneous implantation, G3 orthotopic implantation | 6 weeks | Not reported Lungs, liver | ^44,45^ |
|  |  |  | Patient-derived local recurrence | Athymic nu/nu | G1 subcutaneous implantation, G2 orthotopic implantation | 15 days after size > 100mm^3^ | Not reported | ^36^ |
|  |  | Dedifferentiated liposarcoma | Patient-derived local recurrence | Athymic nu/nu | G1 subcutaneous implantation, G2 orthotopic implantation | 15 days after size > 50mm^3^ | Not reported | ^46–49^ |
|  |  | Myxofibrosarcoma | Unspecified | Athymic nu/nu | G1 subcutaneous implantation, G2 orthotopic implantation | 15 days after size > 70mm^3^ | Not reported | ^50^ |
|  |  | Rhabdomyosarcoma | Primary tumour tissue | Athymic nu/nu | G1 subcutaneous implantation, G2 orthotopic implantation. Tumour resection to evaluate local recurrence. | 12 weeks | Local recurrence | ^51,52^ |
|  |  | Embryonal rhabdomyosarcoma | Lung metastasis | NSG | Submuscular space at the tibia, limb amputation when tumour reached 1,5cm diameter | 20 weeks | Hematogenous and lymphatic spread after amputation | ^7^ |
|  |  | Leiomyosarcoma | Primary tumour tissue | RFP expressing transgenic nude mice | G1 subcutaneous implantation, G3 orthotopic implantation | 14 days post implantation | Not reported | ^53^ |
|  |  |  | Peritoneal metastasis | Athymic nu/nu | G1 subcutaneous implantation, G2 orthotopic implantation | 21 days post implantation | Not reported | ^54^ |
|  |  | Follicular dendritic cell sarcoma | Local recurrence | Athymic nu/nu | G1 subcutaneous implantation, G2 orthotopic implantation | 30 days | Not reported | ^55^ |
|  |  | Synovial sarcoma | Primary tumour tissue | Athymic nu/nu | G1 subcutaneous implantation, G2 orthotopic implantation | 14 days post implantation | Not reported | ^56,57^ |

## Supplementary references

1 Scheinin I, Sie D, Bengtsson H, van de Wiel MA, Olshen AB, van Thuijl HF *et al.* DNA copy number analysis of fresh and formalin-fixed specimens by shallow whole-genome sequencing with identification and exclusion of problematic regions in the genome assembly. *Genome Res* 2014; **24**: 2022–2032.

2 Sante T, Vergult S, Volders P-J, Kloosterman WP, Trooskens G, de Preter K *et al.* ViVar: A Comprehensive Platform for the Analysis and Visualization of Structural Genomic Variation. *PLoS One* 2014; **9**: e113800.

3 Chaves-Urbano B, Hernando B, Garcia MJ, Macintyre G. CNpare: matching DNA copy number profiles. *Bioinformatics* 2022; **38**: 3638–3641.

4 Nanni P, Landuzzi L, Manara MC, Righi A, Nicoletti G, Cristalli C *et al.* Bone sarcoma patient-derived xenografts are faithful and stable preclinical models for molecular and therapeutic investigations. *Sci Rep* 2019; **9**. doi:10.1038/s41598-019-48634-y.

5 Rainusso N, Cleveland H, Hernandez JA, Quintanilla NM, Hicks J, Vasudevan S *et al.* Generation of patient-derived tumor xenografts from percutaneous tumor biopsies in children with bone sarcomas. *Pediatr Blood Cancer* 2019; **66**. doi:10.1002/pbc.27579.

6 Murakami T, Igarashi K, Kawaguchi K, Kiyuna T, Zhang Y, Zhao M *et al.* Tumor-targeting Salmonella typhimurium A1-R regresses an osteosarcoma in a patient-derived xenograft model resistant to a molecular-targeting drug. *Oncotarget* 2017; **8**: 8035–8042.

7 Goldstein SD, Hayashi M, Albert CM, Jackson KW, Loeb DM. An orthotopic xenograft model with survival hindlimb amputation allows investigation of the effect of tumor microenvironment on sarcoma metastasis. *Clin Exp Metastasis* 2015; **32**: 703–715.

8 Blackman G, Pollock RE, Morris R, Harding V, Schwartz GK, Maki R *et al.* Patient-derived xenografts for individualized care in advanced sarcoma. *Cancer* 2014; **120**: 2006–2015.

9 Cornillie J, Wozniak A, Li H, Wang Y, Boeckx B, Gebreyohannes YK *et al.* Establishment and Characterization of Histologically and Molecularly Stable Soft-tissue Sarcoma Xenograft Models for Biological Studies and Preclinical Drug Testing. *Mol Cancer Ther* 2019; **18**: 1168–1178.

10 Laroche A, Chaire V, le Loarer F, Algéo MP, Rey C, Tran K *et al.* Activity of trabectedin and the PARP inhibitor rucaparib in soft-tissue sarcomas. *J Hematol Oncol* 2017; **10**: 1–10.

11 Laroche-Clary A, Chaire V, Verbeke S, Algéo MP, Malykh A, le Loarer F *et al.* ATR Inhibition Broadly Sensitizes Soft-Tissue Sarcoma Cells to Chemotherapy Independent of Alternative Lengthening Telomere (ALT) Status. *Sci Rep* 2020; **10**. doi:10.1038/s41598-020-63294-z.

12 Aoki Y, Yamamoto J, Tome Y, Hamada K, Masaki N, Inubushi S *et al.* Over-methylation of Histone H3 Lysines Is a Common Molecular Change among the Three Major Types of Soft-tissue Sarcoma in Patient-derived Xenograft (PDX) Mouse Models. *Cancer Genomics Proteomics* 2021; **18**: 715–721.

13 Oyama R, Takahashi M, Yoshida A, Sakumoto M, Takai Y, Kito F *et al.* Generation of novel patient-derived CIC-DUX4 sarcoma xenografts and cell lines. *Sci Rep* 2017; **7**. doi:10.1038/s41598-017-04967-0.

14 Cornillie J, Wozniak A, Pokreisz P, Casazza A, Vreys L, Wellens J *et al.* In Vivo Antitumoral Efficacy of PhAc-ALGP-Doxorubicin, an Enzyme-Activated Doxorubicin Prodrug, in Patient-Derived Soft Tissue Sarcoma Xenograft Models. *Mol Cancer Ther* 2017; **16**: 1566–1575.

15 Hiroshima Y, Zhang Y, Zhang N, Uehara F, Maawy A, Murakami T *et al.* Patient-derived orthotopic xenograft nude mouse model of soft tissue sarcoma more closely mimics the patient behavior in contrast to the subcutaneous ectopic model. *Anticancer Res* 2015; **35**: 697–702.

16 Loh AHP, Stewart E, Bradley CL, Chen X, Daryani V, Stewart CF *et al.* Combinatorial Screening Using Orthotopic Patient Derived Xenograft-Expanded Early Phase Cultures of Osteosarcoma Identify Novel Therapeutic Drug Combinations. *Cancer Lett* 2019; : 262–270.

17 Stewart E, Federico SM, Chen X, Shelat AA, Bradley C, Gordon B *et al.* Orthotopic patient-derived xenografts of paediatric solid tumours. *Nature* 2017; **549**: 96–100.

18 Igarashi K, Kawaguchi K, Murakami T, Kiyuna T, Miyake K, Nelson SD *et al.* Intra-arterial administration of tumor-targeting Salmonella typhimurium A1-R regresses a cisplatin-resistant relapsed osteosarcoma in a patient-derived orthotopic xenograft (PDOX) mouse model. *Cell Cycle* 2017; **16**: 1164–1170.

19 Igarashi K, Murakami T, Kawaguchi K, Kiyuna T, Miyake K, Zhang Y *et al.* A patient-derived orthotopic xenograft (PDOX) mouse model of a cisplatinum-resistant osteosarcoma lung metastasis that was sensitive to temozolomide and trabectedin: implications for precision oncology. *Oncotarget* 2017; **8**: 62111–62119.

20 Igarashi K, Kawaguchi K, Kiyuna T, Miyake K, Miyake M, Li S *et al.* Tumor-targeting Salmonella typhimurium A1-R combined with recombinant methioninase and cisplatinum eradicates an osteosarcoma cisplatinum-resistant lung metastasis in a patient-derived orthotopic xenograft (PDOX) mouse model: decoy, trap and kill chemotherapy moves toward the clinic. *Cell Cycle* 2018; **17**: 801–809.

21 Igarashi K, Kawaguchi K, Kiyuna T, Miyake K, Miyake M, Li Y *et al.* Temozolomide combined with irinotecan regresses a cisplatinum-resistant relapsed osteosarcoma in a patient-derived orthotopic xenograft (PDOX) precision-oncology mouse model. 2018.

22 Goldstein SD, Trucco M, Guzman WB, Hayashi M, Loeb DM. A monoclonal antibody against the Wnt signaling inhibitor dickkopf-1 inhibits osteosarcoma metastasis in a preclinical model. *Oncotarget* 2016; **7**: 21114–21123.

23 Miyake K, Murakami T, Kiyuna T, Igarashi K, Kawaguchi K, Miyake M *et al.* The combination of temozolomide-irinotecan regresses a doxorubicin-resistant patient-derived orthotopic xenograft (PDOX) nude-mouse model of recurrent Ewing’s sarcoma with a FUS-ERG fusion and CDKN2A deletion: Direction for third-line patient therapy. 2017.

24 Miyake K, Kiyuna T, Kawaguchi K, Higuchi T, Oshiro H, Zhang Z *et al.* Regorafenib regressed a doxorubicin-resistant Ewing’s sarcoma in a patient-derived orthotopic xenograft (PDOX) nude mouse model. *Cancer Chemother Pharmacol* 2019; **83**: 809–815.

25 Miyake K, Kiyuna T, Li S, Han Q, Tan Y, Zhao M *et al.* Combining Tumor-Selective Bacterial Therapy with Salmonella typhimurium A1-R and Cancer Metabolism Targeting with Oral Recombinant Methioninase Regressed an Ewing’s Sarcoma in a Patient-Derived Orthotopic Xenograft Model. *Chemotherapy* 2019; **63**: 278–283.

26 Murakami T, Singh AS, Kiyuna T, Dry SM, Li Y, James AW *et al.* Effective molecular targeting of CDK4/6 and IGF-1R in a rare FUS-ERG fusion CDKN2A-deletion doxorubicin-resistant Ewing’s sarcoma patient-derived orthotopic xenograft (PDOX) nude-mouse model. *Oncotarget* 2016; **7**: 47556–47564.

27 Murakami T, Li S, Han Q, Tan Y, Kiyuna T, Igarashi K *et al.* Recombinant methioninase effectively targets a Ewing’s sarcoma in a patient-derived orthotopic xenograft (PDOX) nude-mouse model. *Oncotarget* 2017; **8**: 35630–35638.

28 Murakami T, Kiyuna T, Kawaguchi K, Igarashi K, Singh AS, Hiroshima Y *et al.* The irony of highly-effective bacterial therapy of a patient-derived orthotopic xenograft (PDOX) model of Ewing’s sarcoma, which was blocked by Ewing himself 80 years ago. *Cell Cycle* 2017; **16**: 1046–1052.

29 Miyake K, Murakami T, Kiyuna T, Igarashi K, Kawaguchi K, Li Y *et al.* Eribulin regresses a doxorubicin-resistant Ewing’s sarcoma with a FUS-ERG fusion and CDKN2A-deletion in a patient-derived orthotopic xenograft (PDOX) nude mouse model. *J Cell Biochem* 2018; **119**: 967–972.

30 Higuchi T, Kawaguchi K, Miyake K, Oshiro H, Zhang Z, Razmjooei S *et al.* The combination of gemcitabine and nab-paclitaxel as a novel effective treatment strategy for undifferentiated soft-tissue sarcoma in a patient-derived orthotopic xenograft (PDOX) nude-mouse model. *Biomedicine and Pharmacotherapy* 2019; **111**: 835–840.

31 Igarashi K, Kawaguchi K, Li S, Han Q, Tan Y, Murakami T *et al.* Recombinant methioninase in combination with doxorubicin (DOX) overcomes first-line DOX resistance in a patient-derived orthotopic xenograft nude-mouse model of undifferentiated spindle-cell sarcoma. *Cancer Lett* 2018; **417**: 168–173.

32 Igarashi K, Kawaguchi K, Murakami T, Kiyuna T, Miyake K, Singh AS *et al.* High Efficacy of Pazopanib on an Undifferentiated Spindle-Cell Sarcoma Resistant to First-Line Therapy Is Identified With a Patient-Derived Orthotopic Xenograft (PDOX) Nude Mouse Model. *J Cell Biochem* 2017; **118**: 2739–2743.

33 Igarashi K, Kawaguchi K, Kiyuna T, Miyake K, Miyake M, Li Y *et al.* Temozolomide regresses a doxorubicin-resistant undifferentiated spindle-cell sarcoma patient-derived orthotopic xenograft (PDOX): precision-oncology nude-mouse model matching the patient with effective therapy. *J Cell Biochem* 2018; **119**: 6598–6603.

34 Igarashi K, Li S, Han Q, Tan Y, Kawaguchi K, Murakami T *et al.* Growth of doxorubicin-resistant undifferentiated spindle-cell sarcoma PDOX is arrested by metabolic targeting with recombinant methioninase. *J Cell Biochem* 2018; **119**: 3537–3544.

35 Higuchi T, Miyake K, Sugisawa N, Oshiro H, Zhang Z, Razmjooei S *et al.* The combination of olaratumab with gemcitabine and docetaxel arrests a chemotherapy-resistant undifferentiated soft-tissue sarcoma in a patient-derived orthotopic xenograft mouse model. *Cancer Chemother Pharmacol* 2019; **83**: 1075–1082.

36 Kawaguchi K, Igarashi K, Kiyuna T, Miyake K, Miyake M, Murakami T *et al.* Individualized doxorubicin sensitivity testing of undifferentiated soft tissue sarcoma (USTS) in a patient-derived orthotopic xenograft (PDOX) model demonstrates large differences between patients. *Cell Cycle* 2018; **17**: 627–633.

37 Higuchi T, Miyake K, Sugisawa N, Oshiro H, Zhang Z, Razmjooei S *et al.* Olaratumab combined with doxorubicin and ifosfamide overcomes individual doxorubicin and olaratumab resistance of an undifferentiated soft-tissue sarcoma in a PDOX mouse model. *Cancer Lett* 2019; **451**: 122–127.

38 Igarashi K, Kawaguchi K, Murakami T, Kiyuna T, Miyake K, Yamamoto N *et al.* A novel anionic-phosphate-platinum complex effectively targets an undifferentiated pleomorphic sarcoma better than cisplatinum and doxorubicin in a patient-derived orthotopic xenograft (PDOX). *Oncotarget* 2017; **8**: 63353–63359.

39 Igarashi K, Li S, Han Q, Tan Y, Kawaguchi K, Murakami T *et al.* Growth of doxorubicin-resistant undifferentiated spindle-cell sarcoma PDOX is arrested by metabolic targeting with recombinant methioninase. *J Cell Biochem* 2018; **119**: 3537–3544.

40 Kawaguchi K, Igarashi K, Miyake K, Kiyuna T, Miyake M, Singh AS *et al.* Patterns of sensitivity to a panel of drugs are highly individualised for undifferentiated/unclassified soft tissue sarcoma (USTS) in patient-derived orthotopic xenograft (PDOX) nude-mouse models. *J Drug Target* 2019; **27**: 211–216.

41 Igarashi K, Kawaguchi K, Kiyuna T, Miyake K, Miyake M, Li Y *et al.* Temozolomide regresses a doxorubicin-resistant undifferentiated spindle-cell sarcoma patient-derived orthotopic xenograft (PDOX): precision-oncology nude-mouse model matching the patient with effective therapy. *J Cell Biochem* 2018; **119**: 6598–6603.

42 Higuchi T, Miyake K, Sugisawa N, Oshiro H, Zhang Z, Razmjooei S *et al.* The combination of olaratumab with gemcitabine and docetaxel arrests a chemotherapy-resistant undifferentiated soft-tissue sarcoma in a patient-derived orthotopic xenograft mouse model. *Cancer Chemother Pharmacol* 2019; **83**: 1075–1082.

43 Murakami T, Delong J, Eilber FC, Zhao M, Zhang Y, Zhang N *et al.* Tumor-targeting Salmonella typhimurium A1-R in combination with doxorubicin eradicate soft tissue sarcoma in a patient-derived orthotopic xenograft (PDOX) model. *Oncotarget* 2016; **7**: 12783–12790.

44 Oshiro H, Kiyuna T, Tome Y, Miyake K, Kawaguchi K, Higuchi T *et al.* Detection of metastasis in a Patient-Derived Orthotopic Xenograft (PDOX) model of undifferentiated pleomorphic sarcoma with red fluorescent protein. *Anticancer Res* 2019; **39**: 81–85.

45 Kiyuna T, Murakami T, Tome Y, Kawaguchi K, Igarashi K, Miyake K *et al.* Analysis of Stroma Labeling During Multiple Passage of a Sarcoma Imageable Patient-Derived Orthotopic Xenograft (iPDOX) in Red Fluorescent Protein Transgenic Nude Mice. *J Cell Biochem* 2017; **118**: 3367–3371.

46 Kiyuna T, Murakami T, Tome Y, Igarashi K, Kawaguchi K, Miyake K *et al.* Doxorubicin-resistant pleomorphic liposarcoma with PDGFRA gene amplification is targeted and regressed by pazopanib in a patient-derived orthotopic xenograft mouse model. *Tissue Cell* 2018; **53**. doi:10.1016/j.tice.2018.05.010.

47 Igarashi K, Kawaguchi K, Kiyuna T, Miyake K, Miyaki M, Yamamoto N *et al.* Metabolic targeting with recombinant methioninase combined with palbociclib regresses a doxorubicin-resistant dedifferentiated liposarcoma. *Biochem Biophys Res Commun* 2018; **506**: 912–917.

48 Kiyuna T, Tome Y, Murakami T, Zhao M, Miyake K, Igarashi K *et al.* Tumor-targeting Salmonella typhimurium A1-R arrests a doxorubicin-resistant PDGFRA-amplified patient-derived orthotopic xenograft mouse model of pleomorphic liposarcoma. *J Cell Biochem* 2018; **119**: 7827–7833.

49 Kiyuna T, Tome Y, Murakami T, Kawaguchi K, Igarashi K, Miyake K *et al.* Trabectedin arrests a doxorubicin-resistant PDGFRA-activated liposarcoma patient-derived orthotopic xenograft (PDOX) nude mouse model. *BMC Cancer* 2018; **18**. doi:10.1186/s12885-018-4703-0.

50 Kiyuna T, Tome Y, Murakami T, Miyake K, Igarashi K, Kawaguchi K *et al.* A combination of irinotecan/cisplatinum and irinotecan/temozolomide or tumor-targeting Salmonella typhimurium A1-R arrest doxorubicin- and temozolomide-resistant myxofibrosarcoma in a PDOX mouse model. *Biochem Biophys Res Commun* 2018; **505**: 733–739.

51 Igarashi K, Kawaguchi K, Kiyuna T, Murakami T, Miwa S, Nelson SD *et al.* Patient-derived orthotopic xenograft (PDOX) mouse model of adult rhabdomyosarcoma invades and recurs after resection in contrast to the subcutaneous ectopic model. *Cell Cycle* 2017; **16**: 91–94.

52 Igarashi K, Kawaguchi K, Kiyuna T, Murakami T, Miwa S, Nelson SD *et al.* Temozolomide combined with irinotecan caused regression in an adult pleomorphic rhabdomyosarcoma patient-derived orthotopic xenograft (PDOX) nude-mouse model. *Oncotarget* 2017; **8**: 75874–75880.

53 Kawaguchi K, Igarashi K, Murakami T, Kiyuna T, Nelson SD, Dry SM *et al.* Combination of gemcitabine and docetaxel regresses both gastric leiomyosarcoma proliferation and invasion in an imageable patient-derived orthotopic xenograft (iPDOX) model. *Cell Cycle* 2017; **16**: 1063–1069.

54 Miyake K, Kiyuna T, Miyake M, Kawaguchi K, Zhang Z, Wangsiricharoen S *et al.* Gemcitabine combined with docetaxel precisely regressed a recurrent leiomyosarcoma peritoneal metastasis in a patient-derived orthotopic xenograft (PDOX) model. *Biochem Biophys Res Commun* 2019; **509**: 1041–1046.

55 Kiyuna T, Murakami T, Tome Y, Kawaguchi K, Igarashi K, Zhang Y *et al.* High efficacy of tumor-targeting Salmonella typhimurium A1-R on a doxorubicin-and dactolisib-resistant follicular dendritic-cell sarcoma in a patient-derived orthotopic xenograft PDOX nude mouse model. *Oncotarget* 2016; **7**: 33046–33054.

56 Igarashi K, Kawaguchi K, Li S, Han Q, Tan Y, Gainor E *et al.* Recombinant methioninase combined with doxorubicin (DOX) regresses a DOX-resistant synovial sarcoma in a patient-derived orthotopic xenograft (PDOX) mouse model. *Oncotarget* 1926; **9**: 19263–19272.

57 Higuchi T, Kawaguchi K, Miyake K, Han Q, Tan Y, Oshiro H *et al.* Oral recombinant methioninase combined with caffeine and doxorubicin induced regression of a doxorubicin-resistant synovial sarcoma in a PDOX mouse model. *Anticancer Res* 2018; **38**: 5639–5644.
