# Supplementary material for: Post‐operative minimal residual disease models to study metastatic relapse in soft‐tissue sarcoma patient‐derived xenografts
Source: Clin Transl Med. 2023 Jun 6;13(6):e1290. doi: 10.1002/ctm2.1290 (PMC10244893; doi:10.1002/ctm2.1290)
Supplement: Supplementary file 3 — Supporting information [file CTM2-13-e1290-s003.docx]

**Table S2. Patient characteristics.**

| **Patient** | Diagnosis | Sample | Neo-adjuvant therapy | Resection | Adjuvant therapy | FNCLCC | TNM, stage | M+ | DFS | OS |
| --- | --- | --- | --- | --- | --- | --- | --- | --- | --- | --- |
| **MPNST/058** | Malignant peripheral nerve sheath tumour | Biopsy primary tumour | Radiotherapy | R0 | None | 3 | T2N0M0,  IIIA | Yes | 3m | 7m^ⴕ^ |
| **UPS/059** | Undifferentiated pleomorphic sarcoma | Resection primary tumour | Adriamycin-ifosfamide | R0 | Radiotherapy | 3 | T1N0M0,  II | Yes | 6m | >4y |
| **UPS/048/M** | Undifferentiated pleomorphic sarcoma | Metastasis resection | None | R0 | Radiotherapy  Adriamycin-ifosfamide | 3 | T4N0M0, IIIB | Yes | 9m | >4y6m |
| **EOS/045/M** | Extra-skeletal osteosarcoma | Metastasis resection | Euramos protocol | R0 | Euramos protocol | 3 | T2N0M0, IIIA | Yes | 6m | 2y10m^ⴕ^ |
| **MPNST/024** | Malignant peripheral nerve sheath tumour | Resection primary tumour | None | R1 | Radiotherapy | 3 | T2N0M0, IIIA | No | >4y8m | >4y8m |

*Sample* is which sample was used for xenograft development. *Resection* is the completeness of the primary tumour resection according to AJCC 8^th^ edition. *FNCLCC* is the Fédération Nationale des Centres de Lutte Contre Le Cancer (FNCLCC) grading system. *M+* is the development of metastases after tumour resection. *DFS* is disease free survival, *OS* is overall survival, > indicates that the patient is stable and in follow-up. ^ƚ^ indicates that the patient deceased. MPNST/058 is a patient with a primary high-grade malignant peripheral nerve sheath tumour, UPS/059 is a patient with a primary high-grade undifferentiated pleomorphic sarcoma, UPS/048/M is a patient with a metastasis of an undifferentiated pleomorphic sarcoma, EOS/045/M is a patient with a metastasis of an extraskeletal osteosarcoma, MPNST/024 is a patient with a primary high-grade malignant peripheral nerve sheath tumour, m = months, y = years.
