# Supplementary material for: Post‐operative minimal residual disease models to study metastatic relapse in soft‐tissue sarcoma patient‐derived xenografts
Source: Clin Transl Med. 2023 Jun 6;13(6):e1290. doi: 10.1002/ctm2.1290 (PMC10244893; doi:10.1002/ctm2.1290)
Supplement: Supplementary file 4 — Supporting information [file CTM2-13-e1290-s001.docx]

## Supplementary results

### Patient information

MPNST/058
The patient presented with a 5 cm diameter mass at the medial side of the right thigh. Biopsy showed a malignant peripheral nerve sheath tumour, FNCLCC 3, with synchronous detection of a skip metastasis in the right femur. Lung metastases could not be detected at the time of diagnosis. The patient received pre-operative radiotherapy; tumour shrinkage could not be observed. An R0 resection was completed four months after case presentation, anatomopathological review showed a lack of therapy response. Samples for PDX development were collected during biopsy.

Bone metastasis and abdominal metastasis were detected at 3 months after tumour resection. Palliative care was started in agreement with the patient. Overall survival was 7 months.

UPS/059
The patient presented with a 7.5 cm diameter mass on the left upper arm, in close relation to the branchial nerves and artery. Biopsy revealed an undifferentiated high-grade pleomorphic sarcoma, with myogenic lineage differentiation, FNCLCC 3. The patient received neo-adjuvant chemotherapy (doxorubicin-ifosfamide) which had to be ceased after 3 cycles due to tumour growth. The tumour was resected 3 months post-biopsy. Anatomopathological review concluded ypT1N0M0 and minimal resection margins of 2mm (R0), no signs of therapy response could be detected. Adjuvant radiotherapy was given. Samples for PDX development were taken at the time of tumour resection.

At six months post-resection, a growing lesion in the lower lobe of the right lung was detected, for which a wedge resection was performed. Anatomopathological review concluded the diagnosis of sarcoma metastasis. One year and 8 months post-resection, a local recurrence was detected, for which the patient received an upper arm amputation. Currently the patient is 4 years post-diagnosis and disease free.

UPS/048/M
The patient presented with a large mass in the upper left leg (17x8x3 cm) with deep invasion of the musculus quadriceps tendon. Biopsy indicated an undifferentiated pleomorphic sarcoma with myogenic lineage differentiation, FNCLCC 3. Biopsy was followed by R0 resection, adjuvant radiotherapy and chemotherapy (Adriamycin-ifosfamide). The latter had to be terminated prematurely due to multiple neutropenic events.

Nine months post-resection, the patient was diagnosed with 3 lung metastases, successfully treated with wedge resection. Samples for PDX development were harvested during this procedure. Five months later, the patient presented with a new lung nodule, which was again resected. Currently the patient is 3.5 years post-primary tumour resection and has stable disease with no detectable tumour burden.

EOS/045/M
The patient presented with an 8.7 cm diameter mass in the left upper arm, located at the deltoid muscle. A biopsy confirmed the diagnosis of an extraskeletal osteosarcoma, FNCLCC 3. Staging was negative. The patient was treated according to the Euramos protocol, and an R0 resection with extracorporeal radiation was performed. Despite treatment, the patient was diagnosed with metastases in the left lower lobe of the lung 6 months later, for which wedge resection was performed 5 months after radiological detection due to patient refusal. Seven months later, the patient presented again with lung metastasis, for which a lobectomy and radiotherapy were performed. However, in 3 months’ time, multiple lung metastasis as well as positive axillary lymph nodes and peritoneal metastasis were detected, for which antalgic radiotherapy as well as chemotherapy were started. After 3 cycles of Trabectedin, therapy was switched to Gemcitabine-Taxol weekly. The patient deceased due to progressive disease 2 years and 10 months post-diagnosis.

MPNST/024
The patient was diagnosed with a small mass (2x2cm) in the iliopsoas muscle in close relation to radix L3, on a CT scan that was performed in the context of an acute not-related medical problem. Six months later, an MRI was performed, showing growth of the mass (6x6cm), suspect of a high-grade sarcoma. Biopsy showed a malignant peripheral nerve sheath tumour, FNCLCC 3. Staging was negative. The mass was resected 2 weeks after biopsy, confirming the diagnosis. Samples for PDX development were obtained during tumour resection.

Tumour margins were positive (R1 resection) at one site. The patient received adjuvant radiotherapy. The patient is currently 4 years and 8 months in follow-up and disease-free.
