# Supplementary material for: Post‐operative minimal residual disease models to study metastatic relapse in soft‐tissue sarcoma patient‐derived xenografts
Source: Clin Transl Med. 2023 Jun 6;13(6):e1290. doi: 10.1002/ctm2.1290 (PMC10244893; doi:10.1002/ctm2.1290)
Supplement: Supplementary file 5 — Supporting information [file CTM2-13-e1290-s004.docx]

**Table S3. Comparison of patient and PDX metastasis.**

| Code | Patient metastases | PDX metastases |
| --- | --- | --- |
| MPNST/058 | Bone metastasis  Abdominal lymph node metastasis | Bone metastasis  Abdominal lymph node metastasis  Salivary gland metastasis |
| MPNST/024 | None | Lymph node metastasis |
| UPS/059 | Lung metastasis  Local recurrence | Lung metastasis  Bone metastasis |
| UPS/048/M | Lung metastasis | Lung metastasis  Abdominal metastasis |
| EOS/045/M | Lung metastasis  Lymph node metastasis  Retroperitoneal metastasis | Lung metastasis  Lymph node metastasis  Local recurrence |

MPNST/058 is a patient with a primary high-grade malignant peripheral nerve sheath tumour, UPS/059 is a patient with a primary high-grade undifferentiated pleomorphic sarcoma, UPS/048/M is a patient with a metastasis of an undifferentiated pleomorphic sarcoma, EOS/045/M is a patient with a metastasis of an extraskeletal osteosarcoma, MPNST/024 is a patient with a primary high-grade malignant peripheral nerve sheath tumour.
